# Germline *BRCA* testing in routine clinical practice: a single-center experience

**DOI:** 10.3389/pore.2026.1612238

**Published:** 2026-02-20

**Authors:** Aliz Nikolényi, Ágnes Dobi, Dóra Sántha, Renáta Kószó, Máté Iványi, Emese Horváth, Márton Zsolt Enyedi, Katalin Priskin, Bernadett Csányi, Attila Patócs, Henriett Butz, János Papp, Zoltán Varga, Rozália Tóth, Judit Oláh, Zsuzsanna Kahán

**Affiliations:** 1 Department of Oncotherapy, University of Szeged, Szeged, Hungary; 2 Department of Medical Genetics, University of Szeged, Szeged, Hungary; 3 Delta Bio 2000 Ltd., Szeged, Hungary; 4 Department of Molecular Genetics and The National Tumour Biology Laboratory, National Institute of Oncology, Comprehensive Cancer Centre, Budapest, Hungary

**Keywords:** *BRCA1/2*, breast cancer, cancer susceptibility genes, germline testing, medical genetics

## Abstract

The identification of *gBRCA1/2* mutations in breast cancer patients is crucial. Successful identification of the mutations has the potential to alter disease treatment and healthcare management of patients whose relatives harbor pathogenic/likely pathogenic (P/LP) variants. In this retrospective analysis, patient- and disease-specific medical data were analyzed in a cohort of breast cancer patients with a known *gBRCA1/2* status who were treated between 2019–2021. The prevalence and type of *gBRCA1/2* P/LP variants, and their relation to the histopathological data of the cancers, were studied. The presence of one or more clinical criteria leading to germline testing, the outcome of patient management, and family member outcomes were collected. Germline variants were found in 67/259 cases and included 61 P/LP alterations and six “variants of unknown significance” (VUS) of the *BRCA1/2* genes. A spectrum of 31 different variants was detected; eight of them occurred in more than one patient, of which three (detected in 26 cases) belonged to the mutations most prevalently detected by the previously used technology in Hungary. The likelihood of revealing a pathogenic *gBRCA1/2* mutation increased with the number of risk criteria for germline testing. The presence of three or more risk criteria was predictive for carrying a *gBRCA1/2* mutation with an odds ratio (OR) of 10.65 (95% CI 5.20–21.80, p < 0.001). Among the histopathology data, a higher rate of grade 3 or triple negative breast cancer was found among *gBRCA1/2* P/LP variant carriers as compared to that in non-carriers. For ultimately revealing a *gBRCA1/2* P/LP variant, a positive family history (OR 6.69, 95% CI 1.82–24.64, p = 0.003) and triple negative breast cancer (OR 5.65, 95% CI 2.73–11.71, p < 0.001) were the strongest independent predictive factors. Knowing of *gBRCA1/2* alterations meant healthcare management was modified in 86.9% of cases. Germline testing for breast cancer patients, guided by current protocols, is essential for optimizing patient care. Adhering to established clinical criteria facilitates effective patient selection while preventing the unnecessary expansion of testing to average-risk populations. Keywords: *BRCA1/2*, breast cancer, cancer susceptibility genes, germline testing, medical genetics.

## Introduction

Breast cancer is the most common cancer type and a leading cause of death for women worldwide [1]. The majority of breast cancers are sporadic, and the risk of their occurrence is increased by a number of well-known factors such as age, obesity, sedentary lifestyle, smoking, alcohol consumption, hormonal and reproductive history, the diagnosis of certain benign lesions of the breast, or the previous irradiation of the chest [2]. Hereditary breast cancer accounts for only 5%–10% of breast cancers [3] and, in most cases germline mutations of breast cancer, susceptibility genes *BRCA1* and *BRCA2* are responsible for their development [4]. Other uncommon gene variants with high (*TP53, CDH1, PTEN,* and *PALB2*) and low/moderate penetrance (*ATM, CHEK2, BRIP1, BARD1, NBN, NF1, RAD51C, RAD51D,* and *STK11*) also increase breast cancer risk; these variants explain 0%–13% of non-*BRCA1/2-related* hereditary breast cancers, depending on ethnicity [5].

The National Comprehensive Cancer Network (NCCN) Guidelines (v1.2023) recommend testing for high-penetrance breast cancer susceptibility genes in patients diagnosed at age ≤50, those with triple-negative breast cancer (regardless of age), male breast cancer, or Ashkenazi Jewish ancestry. Testing is also indicated for patients with multiple breast cancers (synchronous or metachronous) or a significant family history of associated malignancies. Furthermore, testing guides systemic therapy with PARP inhibitors in both metastatic and adjuvant settings [6]. The detection of cancer susceptibility gene alterations, most importantly *BRCA1* and *BRCA2* mutations, has become of prime interest in recent years for several reasons. The identification of pathogenic *BRCA1* or *BRCA2* carriers prompts the implementation of preventive procedures by means of both primary prevention and screening. In addition to the modification of lifestyle factors that add to genetic risk, prophylactic surgical options, such as risk-reducing mastectomy (RRM) and risk-reducing salpingo-oophorectomy (RRSO), are key interventions in both cancer patients and healthy individuals. Chemoprevention with tamoxifen or anastrozole also seems to be effective in *gBRCA1/2* mutation carriers. Based on a recently published, international, multicenter study of 5,290 *gBRCA1/2* PV-carrier breast cancer patients, after a median follow-up of 8 years, both RRM and RRSO improved overall survival by a HR (Hazard Ratio) = 0.65 (95%CI 0.53–0.78) and HR = 0.58 (95% CI 17.43–18.03), respectively. Although the two interventions were found to be independent, their effects added up [7].

In addition, the oncological treatment of a patient suffering from breast cancer is also directly affected by the detected genetic variation. Genetic testing aids the individualized therapy of breast cancer patients and provides additional treatment options for the affected individuals. In the metastatic setting, both progression-free survival and patient-reported outcome measures were improved in patients treated with the PARP inhibitors olaparib and talazoparib [8, 9]. According to the median 6.1-year follow-up data of the OLYMPIA study, 1 year of adjuvant olaparib therapy significantly improved overall survival, relapse-free survival, and distant disease-free survival in early stage high-risk, HER2-negative breast cancer, irrespective of the hormone receptor status [10]. Furthermore, growing evidence has emerged that incorporation of PARP inhibitor therapy of *gBRCA1/2* PV-carrier breast cancer patients in the neoadjuvant setting may increase the rates of pathological complete response (pCR) [11]. The benefit of platinum-based neoadjuvant chemotherapy in the group of *gBRCA1/2* PV carriers is still controversial: in a randomized INFORM study, no significant difference was demonstrated between doxorubicin vs. platinum-based chemotherapy combinations [12]. In a meta analysis, however, a trend was shown in favor of platinum-based chemotherapy in this specific patient population [13].

In this study, we collected clinicopathological data from breast cancer patients who underwent germline genetic testing and analyzed the prevalence and distribution of gBRCA1/2 mutations within this Hungarian cohort. The aim was to analyze the clinical criteria for predicting the presence of pathogenic germline alterations in these genes and assess the clinical consequences of a positive test result.

## Patients and methods

### Study participants and data collection

A retrospective analysis of a prospectively collected medical database of patients who went through genetic counselling and tested positive for hereditary *BRCA1/2* mutations apropos of their care for breast cancer at the Department of Oncotherapy, University of Szeged, between January 1, 2019, and 1 January 2022, was performed. This study was approved by the Human Investigation Review Board, University of Szeged, Albert Szent-Györgyi Clinical Centre (#160/2020-SZTE). Patients who met at least one of the following criteria were offered genetic testing: 1. Younger than 50 years of age at the time of the diagnosis of breast cancer; 2. Diagnosed with triple negative breast cancer (estrogen receptor -ER/progesterone receptor -PR/human epidermal growth factor receptor 2 -HER2 negative) at the age of <60 years; 3. ≥1 first- or second-degree relatives with breast cancer at the age of ≤50 years; 4. Bilateral breast cancer at the time of the primary diagnosis or later on; 5. Male breast cancer; or 6. Personal history of ovarian cancer. The germline test result of this patient cohort for the *BRCA1/2* genes or a panel of cancer-related genes including *BRCA1/2* could have been obtained at any time point of the disease course within the specified time interval of the study.

Patients undergoing germline genetic testing received mandatory genetic counseling prior to sample collection. These consultations were conducted by clinical geneticists at either the Department of Medical Genetics, University of Szeged, or the National Institute of Oncology, Budapest. According to Hungarian legislation, genetic counselling is the exclusive responsibility of clinical geneticists [14]. The standard procedure includes mandatory pre-test genetic counselling and informed consent being given. Likewise, during a post-test consultation, the result of the molecular genetic test has to be explained to the patient by a genetic counsellor. Genetic counselling for cancer patients primarily focuses on therapeutic implications and recurrence risks. Predictive counselling for healthy individuals from hereditary breast and ovarian cancer families usually involves a broader perspective than therapeutic counselling and usually covers individual risks for other malignancies, descendants’ risk to inherit mutations, preventive options, and lifestyle issues. According to the American College of Medical Genetics and Genomics (ACMG) and European Society for Medical Oncology (ESMO) guidelines [15, 16], medical decisions or predictive testing in relatives at risk are not based on the detection of a VUS.

In the database, the following patient- and tumor-related data were recorded: the patients’ age at the time of the first breast cancer diagnosis; their menopausal status; family history of breast or ovarian tumors; histological type; and grade, ER, PR, and HER2 expression of previously diagnosed breast tumors. ER, PR and HER2 expressions were assessed based on immunohistochemistry (IHC). ER and PR were determined as positive if tumor cell nuclei staining was ≥1%. The tumors were considered HER2 negative if the score was 0 or 1+ and positive if the score was 3+. Fluorescence *in-situ* hybridization (FISH) test was performed and assessed according to the ASCO/CAP HER2 guideline in tumors with IHC 2+ scores. The immunophenotype of the tumor was determined based on ER, PR, and HER2 expression: 1. ER and/or PR positive, HER2 negative; 2. ER and/or PR positive, HER2 positive; 3. ER and PR negative, HER2 positive; and 4. ER, PR, HER2 negative. The stage of the disease at the time of its diagnosis was assessed according to the American Joint Committee on Cancer (AJCC) tumor-node-metastasis (TNM) staging system.

### gBRCA1/2 gene variant detection and analysis

gDNA was isolated from the patients’ peripheral blood samples with the MagCore Genomic DNA Whole Blood Kit (RBC Bioscience). Next-generation sequencing (NGS) library preparation was carried out using a hybridization-based method, targeting *BRCA1/2* genes, including the canonical (+/-30bp) splice donor and acceptor sites and promoter regions (Celemics, Inc.). Sequencing-ready libraries were quality-control checked by Tape Station 4,200 instrument using D5000 ScreenTape (Agilent Technologies USA). NGS was carried out on a NextSeq 550DX sequencing system with NextSeq 500/550 Mid Output Kit v2.5 (300 Cycles) chemistry (Illumina, Inc. United States) or Multiplicom amplicon-based enrichment *BRCA* MASTR Dx or *BRCA* MASTR Plus Dx library preparation kit (Agilent Technologies, Santa Clara, CA) and sequenced on the MiSeq Illumina platform (Illumina, San Diego, CA). Bioinformatics analysis was done with the MASTR Reporter software v.1.1 (Agilent Technologies) and by custom bioinformatic analysis pipeline validated to detect single nucleotid variants (SNV) and small insertion/deletion mutations (<40bp)*. Variants are named according to the HGVS guidelines^1^ using reference sequences NM_007294.4 (BRCA1) and NM_000059.4 (*BRCA2*). The classification of variants follows the guidelines of ENIGMA (Enigma Consortium | Evidence-based Network for the Interpretation of Germline Mutant Alleles^2^). Pathogenic variants were confirmed by Sanger sequencing. Benign or likely benign variants were not reported [17, 18].

The result of the genetic test was documented according to the following nomenclature: 1. *gBRCA1* mutant with a known *BRCA1* pathogenic variant, 2. *gBRCA2* mutant with a known *BRCA2* pathogenic variant, 3. *gBRCA1 or gBRCA2* variant of unknown significance (VUS), or 4. *gBRCA1/2* negative if no *BRCA1/2* pathogenic variant or VUS was detected. These alterations were analyzed in the context of the previously most prevalently detected *gBRCA1/2* mutations in Hungary.

We analyzed the predefined clinicopathological features in the *gBRCA1/2* negative and *gBRCA1/2* mutation carrier cohorts and compared the clinicopathological characteristics of the *gBRCA1* versus *gBRCA2* mutation carrier cohorts. The associations between the clinicopathological characteristics of breast cancers and *gBRCA1/2* status were investigated. We also recorded the type and number of risk factors indicating genetic counselling based on the above listed criteria and analyzed their role in predicting the *gBRCA1/2* mutation carrier status. We evaluated how the result of the genetic test affected the patients' oncological and surgical care and what preventive procedures had been done to reduce cancer risk. In addition, we also recorded the number of cascade *BRCA1/2* screenings in families of patients with a germline *BRCA1/2* mutation and how many of them had tested positive.

### Statistical analyses

Continuous data were expressed as mean ±SD values, if appropriate. Patient- and tumor-related parameters of the *gBRCA1/2* mutation carrier and non-carrier groups were compared with independent sample t-test for the continuous and chi-squared test for the categorical variables. Logistic regression models were applied to evaluate the predictive power of various predefined patient-related factors for the presence of *gBRCA1/2* mutations. First, binary univariate logistic regression models were used separately, followed by a multivariate logistic regression model to examine the joint effects and interactions. A stepwise procedure was used with a likelihood ratio test. The probability of various cancer events in the presence of pathogenic *gBRCA1/2* alterations was examined in a binary univariate logistic regression model. The statistical software IBM SPSS statistics version 29.0 was used for statistical analysis. P-values <0.05 were considered statistically significant.

## Results

A total of 259 patients were included in this study, five of whom were males. Twenty-three additional eligible patients during this time period refused to be tested, while four patients who accepted testing were lost to follow-up; hence, these cases were excluded. *gBRCA1/2* alterations were found in 67 out of 259 cases and included 61 pathogenic alterations (23.55%) and six VUS (2.31%) of the *BRCA1/2* genes; 43/61 (70%) were *gBRCA1* while 18/61 (30%) were g*BRCA2* mutations. No *gBRCA1/2* mutation was found in any of the male patients.

A spectrum of 31 different mutations was detected, 20 in the *BRCA1* and 11 in the *BRCA2* genes (Table 1). Considering all mutations (n = 31), eight occurred in more than one patient, of which three belonged to the *gBRCA* alterations previously highly recurrently detected in the Hungarian population. [19]. The group of eight mutations was present in 38/61 (62.3%) of all *BRCA*1/2 carriers, while founder mutations were detected in 26/61 cases (42.6%).

**TABLE 1:** Types and distribution of pathological gBRCA1/2 variants identified in 61 patients

Gene	Nucleotid change	Amino acid change	Mutation type	Exon	Frequency
BRCA1	c.181T>G	p.(Cys61Gly)	missense	5	14
BRCA2	c.9097dupA	p.(Thr3033AsnfsTer11)	insertion/fs	23	5
BRCA1	c.5266dupC	p.(Gln1756Pro fsTer74)	insertion/fs	20	6
BRCA1	del(ex21-22)	-	deletion	21-22	5
BRCA2	c.9117G>A	p.(Pro3039=)	silent	24	2
BRCA2	c.9371A>T	p.(Asn3124Ile)	missense	25	2
BRCA1	del(ex24)	-	deletion	24	2
BRCA2	c.7913_7917delTTCCT	p.(Phe2638Ter)	nonsense	17	2
BRCA1	c.66_67delAG	p.(Glu23ValfsTer17)	deletion/fs	2	1
BRCA1	c.3700_3704delGTAAA	p.(Val1234GlnfsTer8)	deletion/fs	11	1
BRCA1	c.5278-492_5407-128delins236, del(ex21-22)	p.(Ile1760_Thr1802)	exon deletion	21-22	1
BRCA1	c.5545G>T	p.(Glu1849Ter)	nonsense	24	1
BRCA1	c.5030_5033del CTAA	p.(Thr1677IlefsTer2)	deletion/fs	17	1
BRCA1	c.115T>C	p.(Cys39Arg)	missense	3	1
BRCA1	c.2296_2297delAG	p.(Ser766fsTer)	nonsense	11	1
BRCA1	c.227_228delGT	p.(Ser76AsnfsTer4)	deletion/fs	6	1
BRCA1	c.3756_3759delGTCT	p.(Ser1253ArgfsTer10)	deletion/fs	11	1
BRCA1	c.2329delT	p.(Tyr777MetfsTer15)	deletion/fs	11	1
BRCA1	c.3018_3021delTTCA	p.(His1006GlnfsTer17)	deletion/fs	11	1
BRCA1	dup(ex13)		duplication	13	1
BRCA1	c.1016dupA	p.(Val340GlyfsTer6)	insertion/fs	11	1
BRCA1	c.5251C>T	p.(Arg1751Ter)	nonsense	20	1
BRCA1	c.843_846delCTCA	p.(Ser282TyrfsTer15)	deletion/fs	11	1
BRCA2	c.6656C>G	p.(Ser2219Ter)	nonsense	11	1
BRCA2	c.8755-1G>A		splice acceptor variant	IVS21	1
BRCA2	c.7069_7070delCT	p.(Leu2357ValfsTer2)	deletion/fs	14	1
BRCA2	c.7595_7596insTT	p.(Ala2534LeufsTer18)	insertion/fs	15	1
BRCA2	c.1296_1297delGA	p.(Asn433GlnfsTer18)	deletion/fs	10	1
BRCA2	c.6644_6647delACTC	p.(Tyr2215SerfsTer13)	deletion/fs	11	1
BRCA2	c.9976A>T	p.(Lys3326Ter)	nonsense	27	1
BRCA1	c.5161C>T	p.Gln1721Ter	nonsence	19	1

Due to limited access to genetic testing at our university, these tests had to be performed in two different institutes: the Department of Medical Genetics, University of Szeged, and the Department of Molecular Genetics, National Institute of Oncology, Budapest. In Szeged, testing costs were initially covered by patients out-of-pocket, followed by a private foundation, and subsequently by the National Health Insurance Fund of Hungary. In contrast, all testing in Budapest was funded by the National Health Insurance Fund from the beginning. In 250 cases, genetic tests were restricted to the *BRCA1/2* genes, while in nine cases a comprehensive panel of 113 genes related to hereditary cancers was tested. No difference was found between the number or distribution of pathogenic *BRCA* variants according to the laboratory where the determination was carried out.

The six cases with *BRCA1/2* VUS were excluded from further analyses.

The mean age at the diagnosis of breast cancer of the cohort of 253 patients was 44.6 ± 10.03 (range, 25–78) years.

**FIGURE 1:**
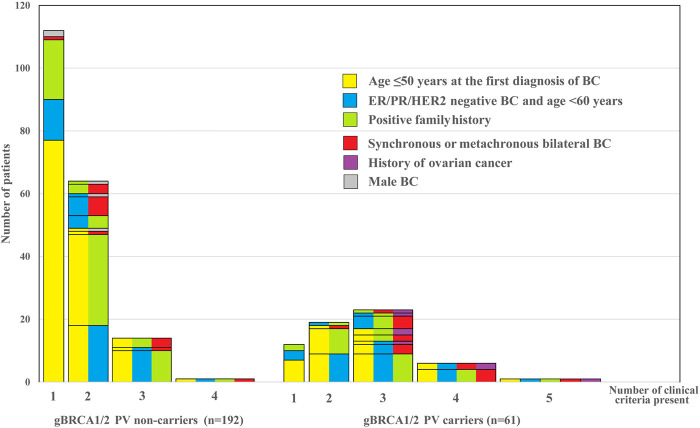
Clinical criteria and their combination suggesting the need of germline testing in a cohort of 253 cases according to the outcome of testing. Bars represent the number of patients with the presence of one or more (1–5) such factors in various combinations according to the test result (pathogenic gBRCA1/2 mutation non-carriers vs. carriers). The color combinations indicate the specific variety of clinical criteria and, cases with identical combinations add up in one rectangle. Note that the bars from left to right in the two groups, respectively, indicate an increasing number of risk criteria that one by one may justify germline genetic testing.

The distribution of cases according to the reason for genetic counselling is represented in Figure 1. While in the *gBRCA1/2* non-carrier group most patients reported one or two clinical criteria only (n = 176; 91.7%), in the *gBRCA1/2* carrier group half of the patients had three or more of them (n = 30; 49.2%) present. The number of risk factors was significantly associated with the finding of *gBRCA1/2* mutation. The presence of three or more risk criteria was predictive for carrying a *gBRCA1/2* mutation with an odds ratio (OR) of 10.65 [95% confidence interval (CI), 5.20–21.80; p < 0.001] (Table 2).

**TABLE 2:** Univariate and multivariate logistic regression analysis of the gBRCA1/2 mutation carrier status and various patient- and tumor-related parameters

Parameter	*BRCA1/2* P/LP non-carriers n = 192 (%)	*BRCA1/2* P/LP carriers n = 61 (%)	OR univariate (95% CI)	OR multivariate (95% CI)
age ≤50 years	141 (73.4)	49 (80.3)	1.48 (0.73-3.01) p=0.280	Excluded
ER/PR/HER2 negative	55 (28.6)	40 (65.6)	4.75 (2.57-8.77) **p < 0.001**	5.65 (2.73-11.71) **p < 0.001**
Positive family history –second degree relatives only	32 (16.7)	10 (16.4%)	1.76 (0.75-4.10) p=0.193	1.47 (0.58-3.72) p=0.422
Positive family history – first degree relatives only	30 (15.6)	21 (34.4)	3.93 (1.90-8.13) **p < 0.001**	5.86 (2.53-13.56) **p < 0.001**
Positive family history – first and second degree relatives	8 (4.2)	6 (9.8)	4.21 (1.33-13.39) **p = 0.015**	6.69 (1.82-24.61) **p = 0.003**
Syncronous or metachronous bilateral breast cancer	16 (8.3)	17 (27.9)	4.25 (1.99-9.07) **p < 0.001**	2.76 (1.17-6.55) **p = 0.021**
Ovarian cancer in personal history	0 (0.0)	7 (11.5)	Excluded	Excluded
Number of risk factors ≥3	16 (8.3)	30 (49.2)	10.65 (5.20-21.80) **p < 0.001**	Excluded

Abbreviations: ER, estrogen receptor; PR, progesteron receptor; HER2, Human Epidermal Growth Factor Receptor 2.

The associations between the clinicopathological variables and the *gBRCA1/2* mutational status of patients are summarized in Table 3. There was no significant difference among the *gBRCA1/2* mutation non-carriers and *gBRCA1/2* mutation carriers regarding the age, proportion of patients under 50 years of age, or menostatus. The histological tumor type did not differ significantly; most of the tumors in both groups were invasive adenocarcinomas of no specific type. The tumor size, nodal status, and tumor stage showed no differences. However, there was a significant difference in the distribution of histopathological grades among the two groups, with a higher rate of grade 3 cancers among the *gBRCA1/2* mutant cases. Likewise, there was a significantly higher rate of triple-negative phenotype in the *gBRCA1/2* mutant cohort. These specific patient- and disease-related characteristics were also investigated according to the type of *BRCA*1/2 mutation. There was no difference regarding the age between the *gBRCA1* versus *gBRCA2* carrier patients, with the mean age at the time of diagnosis being 43.26 ± 8.92 (range, 29–60) versus 41.05 ± 6.70 (range, 28–55) years, respectively. Histopathological grade was higher in cancers of *BRCA1* mutation carriers than that of *BRCA2* mutation carriers. Furthermore, 82.8% of *gBRCA1* mutation carriers had triple-negative breast cancer while that subtype occurred in 5.9% of the *gBRCA2* mutation carriers only. More than two-thirds of the patients carrying a *BRCA2* mutation had hormone receptor-positive and HER2-negative tumors (Table 4).

**TABLE 3:** Patient- and tumor-related characteristics in the entire study population (note that all cancers are included in bilateral breast cancer cases)

Characteristics		*gBRCA1/2* P/LP non-carriers	*gBRCA1/2* P/LP carriers	p
*Patients*		*n* = *192 (%)*	*n* = *61 (%)*	
Mean age (range), years		45.3 (25-78)	42.6 (28-63)	p = 0.068
Age group (%)	<40 years	63 (32.8)	29 (47.5)	p = 0.101
	40–50 years	78 (40.6)	21 (34.4)	
	>50 years	51 (26.6)	11 (18.1)	
Menopausal status/gender (%)	premenopausal	134 (69.8)	49 (80.3)	p = 0.181
	postmenopausal	53 (27.6)	12 (19.7)	
	male	5 (2.6)	0	
*All cancers*		*n* = *208 (%)*	*n* = *78 (%)*	
Breast cancer histologic type	DCIS	12 (5.8)	1 (1.3)	p = 0.141
	NST	163 (78.4)	67 (87.0)	
	ILC	20 (9.6)	3 (3.9)	
	other	13 (6.3)	6 (7.8)	
	not available		1	
TNM tumor size (%)	Tis	12 (5.8)	1 (1.3)	p = 0.365
	T1	73 (35.2)	25 (33.3)	
	T2	91 (44.0)	38 (50.8)	
	T3	23 (11.1)	10 (13.3)	
	T4	8 (3.9)	1 (1.3)	
	not available	1	3	
TNM nodal status (%)	N0	124 (60.2)	46 (61.4)	p = 0.255
	N1	68 (33.0)	21 (28.0)	
	N2	8 (3.9)	7 (9.3)	
	N3	6 (2.9)	1 (1.3)	
	not available	2	3	
TNM stage (%)	Stage 0	12 (5.8)	1 (1.3)	p = 0.059
	Stage 1	57 (27.7)	19 (24.4)	
	Stage 2	103 (50.0)	34 (43.6)	
	Stage 3	20 (9.7)	13 (16.7)	
	Stage 4	14 (6.8)	7 (9.0)	
	not known	2	4	
*Invasive cancers*		*n* = *196 (%)*	*n* = *77 (%)*	
Histological grade	1	18 (9.4)	1 (1.4)	p = 0.006
	2	72 (37.7)	18 (24.6)	
	3	101 (52.9)	54 (74.0)	
	not available	5	4	
IHC subtype groups (%)	ER and/or PR+, HER2-	115 (58.7)	23 (30.7)	<0.001
	ER and/or PR+, HER2+	16 (8.2)	1 (1.3)	
	ER and PR-, HER2+	7 (3.6)	2 (2.7)	
	ER and PR-, HER2-	58 (29.6)	49 (65.3)	
	not available		2	

Abbreviations: DCIS, ductal carcinoma in situ; ER, estrogen receptor; HER2, Human Epidermal Growth Factor Receptor 2; IHC, immunohistochemistry; ILC, invasive lobular cancer; NST, adenocarcinoma of the breast of no special type; PR, progesteron receptor; TNM, tumor-node-metastasis.

**TABLE 4:** Selected patient- and tumor-related characteristics according to the gBRCA1 versus gBRCA2 P/LP carrier status (note that in bilateral cases all cancers are included independently of the time of diagnosis during the course of the disease).

Characteristics		*gBRCA1* P/LP carriers n=43 (%)	*gBRCA2* P/LP carriers n=18 (%)	
Mean age ± SD, years		43.26 ± 8.92	41.05 ± 6.70	**p = 0.352**
*Invasive cancers*		*n = 59 (%)*	*n = 18 (%)*	
Histological grade (%)	1	0 (0)	1 (6.3)	**p = 0.065**
	2	12 (21.1)	6 (37.5)	
	3	45 (78.9)	9 (56.2)	
	not available	2	2	
IHC subtype, (invasive cancers,%)	ER and/or PR+, HER2-	8 (13.8)	15 (88.2)	**p < 0.001**
	ER and PR-, HER2+	1 (1.7)	1 (5.9)	
	ER and/or PR+, HER2+	1 (1.7)	0 (0.0)	
	ER and PR-, HER2-	48 (82.8)	1 (5.9)	
	not available	1	1	

Abbreviations: ER, estrogen receptor; HER2, Human Epidermal Growth Factor Receptor 2; IHC, immunohistochemistry; PR, progesteron receptor.

Next, we evaluated the association between the indications for genetic testing and the resulting genetic findings. A history of breast cancer in first-degree relatives exclusively was elicited from 51 (20.2%) patients. Another 42 patients (16.6%) reported a second-degree relative without affected first-degree relatives. In addition, 14 patients had breast cancer in both first-degree and second-degree relatives. The incidence of *gBRCA1/2* mutation was the highest among the patients with a personal history of ovarian cancer (7/7, 100%), followed by the occurrence of a synchronous bilateral breast cancer (SBBC) or metachronous bilateral breast cancer (MBBC) (17/33, 51.5%). A family history of breast cancer in first- and second-degree relatives (6/14, 42.9%) or in first-degree relatives exclusively (21/51, 41.2%), as well as the triple-negative phenotype of breast cancer (40/95, 42.1%), also indicated a high prevalence of *gBRCA1/2* mutation. Further analyses were performed in logistic regression models. In univariate analysis, the strongest predictive effect on the detection of a pathogenic *gBRCA1/2* mutation was shown for a triple-negative phenotype with an OR of 4.75 (95% CI 2.57–8.77, p < 0.001) and the presence of ≥3 predefined factors with an OR of 10.65 (95% CI 5.20–21.80, p < 0.001) (Table 2). In multivariate analysis, a positive family history (OR 6.69, 95% CI 1.82–24.61, p = 0.003), triple-negative breast cancer (OR 5.65, 95% CI 2.73–11.71, p < 0.001), and bilateral breast cancer (OR 2.76, 95% 1.17–6.55, p = 0.021) remained independent predictive factors (Table 2).

Knowing of gBRCA1/2 alterations meant healthcare management was modified in 43/61 (70.5%) of cases for one or more of the following aspects: a platinum agent was integrated into the neoadjuvant chemotherapy regimen in 12 cases, the adjuvant PARP inhibitor olaparib therapy was recommended for two patients, while 13 patients subsequently received palliative PARP inhibitor therapy for metastatic disease. The original surgical plan was revised by the result of the genetic test for 13 patients (mastectomy was performed instead of excision). Altogether 13 patients underwent risk-reducing contralateral mastectomy, and 16 patients made a decision on prophylactic salpingo-oophorectomy. With regard to the relatives, cascade *BRCA1/2* screening was performed in 25 families; in 19 families, 25 germline pathogenic variants were identified. There were only 10 cases (16.4%) in which the knowledge of the *gBRCA1/2* PV carrier status neither influenced the systemic therapy nor led to prophylactic surgery or prompted a cascade screening of the family.

## Discussion

In this retrospective study, we analyzed the initial implementation of genetic testing in routine clinical practice. Among 259 cases preselected based on high-risk indicators, we identified a 23.5% rate of pathogenic mutations. We evaluated the associations between the clinicopathological features and the ultimate finding of *gBRCA1* or *gBRCA2* mutations: a second ovarian cancer, the diagnosis of SBBC or MBBC, or the simultaneous presence of multiple clinical criteria besides having a triple negative breast cancer phenotype at the age of <60 years or a positive family history were identified as the strongest predictive factors.

The identification of gBRCA1/2 mutations in breast cancer patients is fundamental. Successful identification of the mutations has the potential to alter disease treatment and the future healthcare management of patients whose relatives harbor pathogenic/likely pathogenic (P/LP) variants. A cascade screening extended to family members may play a significant role in cancer prevention [20]. The reception of germline genetic testing has immensely changed in the past few years. Both the evolution of professional guidelines and increased openness from the side of patients and society have occurred. True accelerator factors for these developments have been the registration of the targeted agent PARP inhibitors 8 and 3 years ago for metastatic and early breast cancer, respectively, together with the widening access to genomic testing and the “Angelina Jolie effect” [21].

Current NCCN guidelines recommend testing for high-penetrance breast cancer susceptibility genes in all patients diagnosed at age ≤50. Testing is also indicated to guide systemic therapy with PARP inhibitors or regardless of age in cases of triple-negative breast cancer, multiple primary breast cancers, or a positive family history [20]. The ASCO guideline suggests an age limit of ≤65 years for universal testing of all breast cancer patients with stage 1–4 disease and the examination of older patients if any positive impact could be expected on their systemic therapy or if they had triple negative cancer, a positive family history, an Ashkenazi Jewish ancestry, or if they were males [22]. Finally, the American Society of Breast Surgeons recommends testing of at least *gBRCA1/2* and *gPALB2* (but a larger panel of genes if the alteration of other genes is suspected) in all breast cancer cases [23]. Notably, a significant prevalence of germline alterations was detected among nearly 1,000 breast cancer patients using a wide panel who did not meet screening criteria [24]. In fact, the global limitations in clinical geneticist and laboratory capacities at present restrict the extension of service to all breast cancer patients. The use of comprehensive gene panels would render even more burden on that insufficient network by revealing at least double the pathogenic alterations, although with lower penetrance than that of the *gBRCA1/2* gene mutations [25, 26]. Another consequence that would encumber both the individual and the healthcare system is the identification of VUS that must be followed for its correct evaluation in view of newly evolving data and experiences. Nevertheless, efforts made in this field will result in better survival of mutation carriers: targeted breast magnetic resonance imaging (MRI) screening results in improved survival among healthy individuals carrying *gBRCA*1/2 P/LP variants [27] and breast cancer-specific mortality is significantly improved if genetic testing is performed before the occurrence of breast cancer [28]. Moreover, *gBRCA1/2* mutant breast cancer patients show improved survival if they undergo a risk-reducing mastectomy and/or bilateral salpingo-oophorectomy [7]. The use of PARP inhibitors improves survival as well [8, 29, 30]. It is predicted that these various interventions based on the knowledge of high-penetrance breast cancer susceptibility gene mutation carrier status will be increasingly utilized in both breast cancer and high-risk healthy individuals. It has been demonstrated that both the use of comprehensive cancer gene panels (MGP) and extending testing to a wider population renders this service more cost-effective [31], even in Hungary, where the largest genomic center showed that using a multigene panel testing decreased both the cost and turnaround time significantly [32].

In 2018, we introduced systematic genetic testing for at-risk individuals, implementing the NCCN guidelines for patient selection that were in effect at that time. Although initially we were faced with many difficulties, now all patients or healthy individuals in need in Hungary have potential access to genetic testing with varying turnaround times. Patients have recently become more knowledgeable, probably due to media and informal communication: while initially some patients refused examination, nowadays most patients actively seek genetic testing. The correct information and interpretation of findings by the multidisciplinary team and sufficient time for decision making are key elements for correct patient management. The findings from the Surveillance, Epidemiology, and End Results (SEER) database analysis by Kurian et al. increased attention to a trend of care less aligned with guidelines (i.e., less radiotherapy and more chemotherapies were delivered) of breast cancer patients who carried a pathologic germline mutation as opposed to non-carriers, even if a less penetrant alteration was present [33]. We believe that the practice of germline breast cancer susceptibility gene testing is rapidly evolving and both providers and consumers will make an impact on it. At present, much could be gained through the provision of systematic screening of high-risk breast cancer individuals with no gaps in access.

Universal testing for all breast cancer patients, regardless of clinical data or family history, is an emerging approach that increases both testing access and the detection rate of PVs. Two robust microsimulation studies, one using UK and US databases [34] and the other just US databases [35], have demonstrated that the universal testing of *BRCA1/2* and *PALB2* in breast cancer patients have major effects on cancer prevention and treatment and is extremely cost-effective. A rapid transformation of routine practice, including the widening of the patient population tested and genes examined, is foreseen in the future.

Among our high-risk patients, the prevalence of *gBRCA1* and *gBRCA2* mutations was 16.6% and 6.9%, respectively. In a similar study conducted in Romania in a cohort satisfying the international criteria for testing, similar rates of 12% and 6.8% of *gBRCA1* and *gBRCA2* mutations were found [36]. Likewise, in a recently published Turkish paper, 13.6% *gBRCA*1 and 12.3% *gBRCA2* mutation rates were detected [37]. In a recent Hungarian prospective study analyzing 463 patients, Nagy et al. showed that 96 patients (20.7%) harbored P/LP variants, 67 in high-penetrance genes (56 in *BRCA*1/2) and 29 in moderate-penetrance genes. P/LP variants in this cohort belonged to the *BRCA1* and *BRCA2* genes in 6.9% and 5.2%, respectively. The use of an extended breast cancer panel doubled the detection rate from 12.1% (56/463) to 20.7% (96/463) as compared to *BRCA1/2* testing only [38].

According to literature data, 4%–40% of men diagnosed with breast cancer harbor a *gBRCA2* mutation, while the detection of a *gBRCA1* PV is much rarer; the presence of *gBRCA2* PV indicates a lifetime risk for breast cancer of 8.9% [39, 40]. Notably, due to the lack of large studies, no exact prevalence data are known. In this specific group, the median age at diagnosis is 62 years, most of the cancers are of high grade, ER, PR positive, and HER2 negative, and the disease is of poor outcome; in contrast with their female counterparts, the lobular histology is unusual [40]. Although the described age and histology features were typical among our male breast cancer patients, no *gBRCA1/2* mutation was detected among them [39]. We think that the insufficient case number led to the absence of *gBRCA1/2* PV among our male breast cancer patients. In fact, we had 11 male breast cancer patients whom we offered germline genetic testing to during the time period specified, however, only five patients participated. Our findings are consistent with those of Cheng et al., who recently reported lower rates of genetic testing compliance compared to female patients.

In this research, only 26/61 (42.6%) of the *gBRCA1/2* variants belonged to the well-known, recurrent pathogenic alterations for Hungary, demonstrating the clinical relevance of comprehensive full-length testing of the *BRCA1* and *BRCA2* genes over the testing of hotspot regions. In our study, although the proportion of patients <40 years of age was higher in *gBRCA1/2* mutation carriers versus non-carriers (47.5% versus 32.8%), there was no difference between the mean age of the two groups. One explanation for this finding could be that a young age at diagnosis itself was an inclusion criterion. Notably, by performing systematic germline breast cancer gene alteration screening, various pathological variants including that of the *BRCA1/2* genes were revealed in older patients too [25]. Penetrance of the disease, and differences among ethnicities and races, may also have an impact [41–43].

There were no significant differences between the *gBRCA1/2* carriers and non-carriers regarding the cancer-related data. Most studies have reported that the *gBRCA* mutation status has no influence on tumor size or lymph node status [37, 44–48]. Nonetheless, some studies reported larger tumor sizes and a higher percentage of lymph node metastases in *gBRCA* mutation carriers [49–51]. Regarding the histological type, several studies have confirmed that the majority of invasive breast cancers are breast adenocarcinoma of no special type; a higher frequency of lobular and tubular cancers were reported in *gBRCA2*-associated tumors [52, 53]. Yadav et al, specifically focusing on ILC, found that pathological variants in the *ATM*, *BRCA2*, *CDH1*, *CHEK2*, and *PALB2* genes increased the risk of that histological type while those of the g*BRCA1* gene did not [54]. In line with this finding, our three ILC patients in the *gBRCA1/2* group carried the alteration of the *BRCA2* gene.

Tumors arising in *gBRCA1* mutation carriers are more often poorly differentiated and have a higher histopathological grade than those with a *gBRCA2* mutation or without *gBRCA1/2* mutations [37, 44, 55, 56]. We also found that the grade was higher in *gBRCA1*-related breast cancers than in *gBRCA2*-related breast cancers and *gBRCA1/2* mutation non-carriers.

Triple-negative breast cancer represents 10%–15% of sporadic invasive breast carcinomas. Nevertheless, among *gBRCA1* and *gBRCA2* mutation carriers, 50%–88% and 14.6%–34% of the breast tumors were triple negative, respectively [37, 55, 57]. Our results are in accordance with these findings. Although genetic testing was recommended for triple-negative breast cancer patients with age restriction during the time period we studied, updated guidelines suggest the indication criteria for genetic counselling should be expanded to include all triple-negative breast cancer patients, regardless of age. Although no ER-low positive breast cancers were identified in our study, we consider it important to emphasize that these tumors are more similar to triple-negative breast cancers in terms of both their clinical behavior and genetic characteristics. According to data from the literature, the proportion of *gBRCA1/2* PV carriers among patients with ER-low positive tumors is comparable to that observed in the triple-negative subgroup; hence, genetic testing is considered justified in ER-low cases [58].

Our findings support that, beyond positive family history, bilateral breast cancer, and diagnosis of a second ovarian tumor, specific pathological features of the breast tumor may serve as valuable indicators in identifying patients at elevated risk for hereditary breast cancer. Our results confirm that the aspects indicating genetic counseling, as defined by international guidelines, are associated with a higher likelihood of identifying hereditary genetic alterations as a risk factor for breast cancer compared to an unselected breast cancer population, thereby supporting the cost-effective implementation of genetic testing in routine clinical practice.

## Conclusion

The germline testing of breast cancer patients based on actual guidelines is necessary for optimizing the care of related individuals; considering well-established clinical criteria both promotes patient selection for genetic testing and prevents the unnecessary testing of broader populations of average risk.

## Data Availability

The raw data supporting the conclusions of this article will be made available by the authors, without undue reservation.

## Footnotes

1
http//www.hgvs.org

2
https://enigmaconsortium.org/
